# Areas of Convergence and Divergence in Adolescent Social Isolation and Binge Drinking: A Review

**DOI:** 10.3389/fnbeh.2022.859239

**Published:** 2022-03-30

**Authors:** Jyoti Lodha, Emily Brocato, Jennifer T. Wolstenholme

**Affiliations:** ^1^Department of Pharmacology and Toxicology, Virginia Commonwealth University, Richmond, VA, United States; ^2^VCU Alcohol Research Center, Virginia Commonwealth University, Richmond, VA, United States

**Keywords:** adolescent, social isolation stress, binge ethanol, dendritic spine, myelin, cognitive behavior, social behavior

## Abstract

Adolescence is a critical developmental period characterized by enhanced social interactions, ongoing development of the frontal cortex and maturation of synaptic connections throughout the brain. Adolescents spend more time interacting with peers than any other age group and display heightened reward sensitivity, impulsivity and diminished inhibitory self-control, which contribute to increased risky behaviors, including the initiation and progression of alcohol use. Compared to adults, adolescents are less susceptible to the negative effects of ethanol, but are more susceptible to the negative effects of stress, particularly social stress. Juvenile exposure to social isolation or binge ethanol disrupts synaptic connections, dendritic spine morphology, and myelin remodeling in the frontal cortex. These structural effects may underlie the behavioral and cognitive deficits seen later in life, including social and memory deficits, increased anxiety-like behavior and risk for alcohol use disorders (AUD). Although the alcohol and social stress fields are actively investigating the mechanisms through which these effects occur, significant gaps in our understanding exist, particularly in the intersection of the two fields. This review will highlight the areas of convergence and divergence in the fields of adolescent social stress and ethanol exposure. We will focus on how ethanol exposure or social isolation stress can impact the development of the frontal cortex and lead to lasting behavioral changes in adulthood. We call attention to the need for more mechanistic studies and the inclusion of the evaluation of sex differences in these molecular, structural, and behavioral responses.

## Introduction

Alcohol is the most used and misused substance among adolescents (SAMHSA, 2019). Underage drinking is common and frequently occurs as binge drinking, which is defined as consuming more than 4–5 drinks on the same occasion (SAMHSA, 2019). According to the 2019 SAMHSA report, more than 7 million 12–20-year-olds reported drinking in the past 30 days. Of those, over 4 million reported binge drinking and almost 1 million reported heavy alcohol use, defined as drinking on five or more days in the past month (SAMHSA, 2019). In 2019, the annual Monitoring the Future report stated that by their senior year of high school, more than 50% of teens reported alcohol use in the past year (Miech et al., 2020). Adolescents respond differently to alcohol than their adult counterparts, showing increased sensitivity to alcohol’s rewarding aspects, and decreased sensitivity to the aversive aspects of alcohol (Spear, 2014). This may enable adolescents to increase their alcohol consumption, giving rise to the binge drinking behavior commonly seen among this age group.

Drinking at an early age increases the risk for alcohol use disorders (AUD) later in life, leads to long-term cognitive and behavioral problems, and induces structural changes in the brain (Grant and Dawson, 1997; Spear, 2000, 2018). The risk of developing adult AUD increases the younger one first engages in alcohol consumption (Grant, 1998; Merikangas et al., 1998). Individuals who begin drinking at age 15 are about four times more at risk for developing an AUD than individuals who begin drinking after age 21 (Grant and Dawson, 1997). Adolescent alcohol use is also associated with a number of cognitive problems including deficits in attention and executive function (Spear, 2018; Lannoy et al., 2019; Lees et al., 2020). Verbal learning and short-delay memory tasks were impaired in adolescents that exhibited extreme binge drinking, consuming 10+ drinks per occasion at least once (Nguyen-Louie et al., 2015). Short and long term verbal recall (Parada et al., 2011; Mota et al., 2013) and visuospatial memory impairments (Hanson et al., 2011b) have been found in drinking adolescents, some of which have persisted during abstinence (Hanson et al., 2011a; Carbia et al., 2017). Persistent changes in cortical and subcortical structures are also reported following initiation and continued binge drinking during adolescence (De Bellis et al., 2005; Medina et al., 2008; McQueeny et al., 2009; Bava et al., 2013; Squeglia et al., 2015; Pfefferbaum et al., 2018). Adolescent brain development is characterized by increases in myelination and synaptic pruning, both of which are negatively impacted by alcohol use (Arain et al., 2013; Lees et al., 2020). In a recent study of adolescent drinkers, any alcohol use was associated with accelerated gray matter reductions in frontal and cingulate cortices and subcortical structures (i.e., amygdala, caudate, and hippocampus), regions involved in reward and memory (El Marroun et al., 2021). White matter is also impacted by adolescent alcohol use, causing decreases in white matter volume (De Bellis et al., 2005; Medina et al., 2008; Pfefferbaum et al., 2018) and changes to myelin integrity (Bava et al., 2013). Binge ethanol consumption in adolescent rodents leads to similar decreases in myelination (Montesinos et al., 2014; Vargas et al., 2014; Vetreno et al., 2016; Wolstenholme et al., 2017; Tavares et al., 2019), impaired structural integrity of white matter in the frontal cortex, alterations in dendritic morphology, and cognitive problems in adulthood.

**FIGURE 1:**
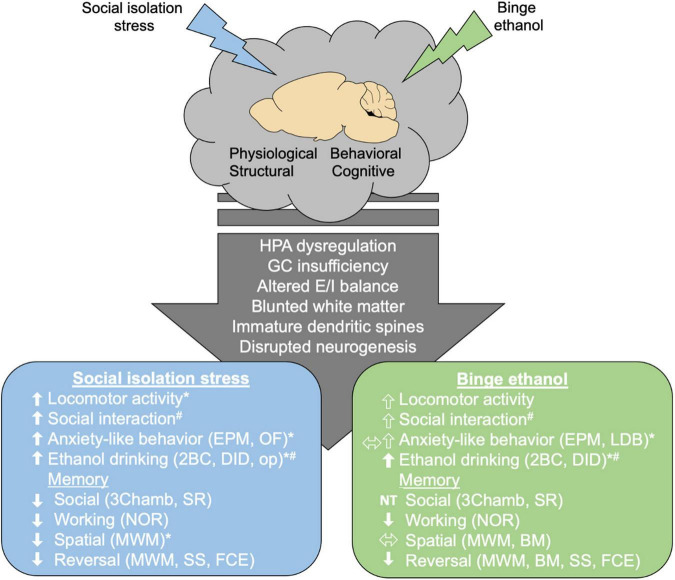
Effects of social isolation stress or binge ethanol in adolescence. The adolescent period is characterized by physiological and structural changes that help mature brain connectivity leading to adult typical behavior and cognition. Postweaning social isolation or binge ethanol in adolescence (∼PND 28–50) alters HPA function, brain connectivity, and generally retains adolescent phenotype (decreased white matter and improper dendritic spine pruning). As reviewed, social isolation stress or binge ethanol increased social interaction, anxiety-like behavior and ethanol drinking. Memory deficits were found following both paradigms. * Indicates conflicting findings in some studies. ^#^ Indicates sex differences were reported. Arrow outlines indicate that the effect depends if acute ethanol is used. HPA, hypothalamic pituitary adrenal axis; GC, glucocorticoid; E/I, excitatory/inhibitory; EPM, elevated plus maze; OF, open field; LDB, light dark box; 2BC, 2-bottle choice; DID, drinking in the dark; op, operant responding; 3chamb, 3-chamber social interaction task; SR, social recognition; NOR, novel object recognition; MWM, Morris water maze; BM, Barnes maze; SS, set shifting; FCE, fear conditioned extinction.

Stressful early life events are associated with problematic underage drinking and alcohol misuse later in life (Dube et al., 2002; Zaso et al., 2021). In heavy drinking college students, drinking alone was associated with more alcohol-related problems, increased feelings of loneliness, and a higher tendency of drinking to cope with stress (Gonzalez et al., 2009; Gonzalez and Skewes, 2013). Feelings of social isolation or perceived burdensomeness, low social support, and greater drinking problems (i.e., higher AUDIT scores) all share similar connectivity patterns which differed from controls (Gonzalez et al., 2009). Low stress resilience (emotional instability, heightened aggression, adjustment difficulties) in adolescent males correlated with increased risk of alcohol consumption and dependence, along with increased use of addictive substances (Kennedy et al., 2019). This link between early life social stress and alcohol use calls for a more thorough investigation of how social stress may worsen drinking outcomes. Animal studies are beginning to elucidate the roles of adolescent binge drinking and social isolation stress in brain connectivity and behavior, specifically in the domains of social anxiety, cognition, and increased risk for adult alcohol misuse (Figure 1). However, a majority of these studies have looked at binge drinking and social isolation stress separately, leaving the combinatorial effects of early life stress and binge drinking largely unexplored. Despite considerable overlap between the behavioral and structural consequences of adolescent binge ethanol and social isolation stress, some behavioral tasks may show opposing effects in adolescents subjected to binge ethanol versus social isolation. Here, we review the behavioral and structural consequences of social isolation or binge drinking in adolescents. As the combinatorial effects of social isolation and binge drinking are not as well documented, we highlight these gaps in the literature and call for more mechanistic studies to understand the effects of social isolation on increased risk of alcohol misuse in adulthood.

### Adolescence Is a Critical Period for Frontal Cortex Brain Development and Maturation

Adolescence, the period of ∼12–20 years old in humans and from weaning [postnatal day (PND) 21] to ∼8 weeks in rodents, is characterized by high levels of playful social interactions, cognitive development, and increased risk-taking behaviors (Spear, 2000). During this period, brain volume and white matter increase linearly with age (Giedd et al., 1999; Sowell et al., 2002; Giedd, 2004) until age 20 (Pfefferbaum et al., 1994). In the prefrontal cortex (PFC), neurons also undergo dramatic structural reorganization and synaptic remodeling (Giedd, 2004). In the prefrontal and orbitofrontal cortexes, up to 50% of spines are pruned from early to mid-adolescence (Bourgeois et al., 1994; Huttenlocher and Dabholkar, 1997; Shapiro et al., 2017). Simultaneously, synaptic connections are being strengthened and refined in an activity-dependent manner (Shapiro et al., 2017; Hinton et al., 2019). In addition to changes in white matter and synaptic refinement, a shift in connectivity among mesocortical and mesolimbic regions occurs (Spear, 2015) with increased connectivity between limbic regions and cortical regions taking place (Yetnikoff et al., 2014). This increasing top–down control from cortical regions during the transition from adolescence to adulthood is associated with greater planning, behavioral flexibility, and executive control (Munakata et al., 2012). Cognitive changes in the developing adolescent include heightened reward sensitivity, sensation seeking, impulsivity, and diminished inhibitory self-control, all of which contribute to increased participation in risky behaviors, including the initiation and escalation of alcohol use (Spear, 2000; Lees et al., 2020). Binge drinking can be particularly harmful in adolescence as ethanol may delay or disrupt critical ongoing neurodevelopment, with profound consequences in adult behaviors, brain structure, connection, and function (De Bellis et al., 2005; Medina et al., 2008; Bava et al., 2013; Jacobus et al., 2013).

Beginning in adolescence, males and females switch their primary focus from family centered relationships to peer relationships (Nelson et al., 2005). In humans and rodents, adolescents share a marked increase in social interactions that serve as a learning experience and shape adult behaviors (van Kerkhof et al., 2013). Peer to peer interactions peak during adolescence and are thought to be crucial for social and cognitive development (Vanderschuren et al., 1997; Spinka et al., 2001). Adolescent rats spend more time engaged in social interactions and social play, and find social experiences more rewarding than adult rats (Trezza et al., 2010; Spear, 2011). Compared to adults, social interactions have a greater impact on decision making and behavior of adolescents (Gardner and Steinberg, 2005). Beneficial and adverse social experiences during adolescence influence anxiety-like (Weiss et al., 2004; Voikar et al., 2005; Koike et al., 2009; McCool and Chappell, 2009; Makinodan et al., 2012; Chappell et al., 2013; Yorgason et al., 2013; Skelly et al., 2015; Lander et al., 2017) and depressive-like behavior (Koike et al., 2009; Amiri et al., 2015), as well as cognition in adulthood (Koike et al., 2009; Zhang et al., 2016; Cao et al., 2017; Lander et al., 2017; Pais et al., 2019). Social isolation can be particularly disruptive at this time as it alters proper neural development and function (Arain et al., 2013; Butler et al., 2016) and increases risk for alcohol use (Lopez et al., 2011; Sanna et al., 2011). Thus, the age at which an individual is deprived of social interactions is critical for ongoing brain and behavioral development.

During adolescence, mesocortical connectivity increases and begins to exert top-down control of limbic regions (Spear, 2015). Adolescents have fewer mesocortical dopamine (DA) fibers, but similar levels in mesolimbic areas than adults (Naneix et al., 2012), indicating a developmental difference in the maturation process of these regions. As mesocortical signaling strengthens, higher cortical control of behavior occurs as the brain transitions from adolescence into adulthood.

Broadly, organizational hormonal actions and hormone receptor expression seems to drive region-specific sex differences in the hippocampus, amygdala, and prefrontal cortex, with females maturing slightly faster than males (Premachandran et al., 2020). Male rodents have larger corticolimbic hippocampal, amygdala, and PFC volumes than females, driven by the size of specific subregions within these structures. Several studies have also shown that the GABA switch from excitatory to inhibitory neurotransmission occurs a few days earlier in females, giving males a longer window of excitatory GABAergic activity in the hippocampus (Premachandran et al., 2020). Though sex differences are less investigated in the PFC, limited evidence suggests that gonadal hormones induce changes in GABAergic maturation, a major factor in the development of the excitatory/inhibitory balance of the PFC (Drzewiecki et al., 2020; Premachandran et al., 2020). Little is known about when sex differences occur in amygdala GABA signaling, only that its transmission is mediated by estrogen (Premachandran et al., 2020). Sex differences in dendritic spine development vary between subregions in the corticolimbic system, particularly the PFC, likely due to external excitatory inputs and GABA maturation (Premachandran et al., 2020). There are limited studies regarding transient sex differences in dopamine circuitry development, but they suggest DA fibers innervating the prelimbic and infralimbic layers of the PFC differ between males and females at PND 7 (Lolier and Wagner, 2021), but not after PND 25 (Willing et al., 2017; Premachandran et al., 2020). Gaps in the developmental time course and dearth of female subjects makes pinpointing the duration of sex-specific critical periods difficult during the maturation of the corticolimbic system (see Premachandran et al., 2020 for more detail) and highlight the need for further studies investigating such differences.

Disruptions to these systems by social isolation and/or binge ethanol can stunt cortical development and maintain use of the more juvenile limbic system (Crews et al., 2016). Juvenile social isolation or social stress leads to structural and molecular changes throughout the brain including reduced neuronal excitability in the dentate gyrus (Sanna et al., 2011; Talani et al., 2016), enhanced DA and serotonin (5-HT) function in the basal ganglia (Yorgason et al., 2013, 2016), alterations in glutamatergic and GABAergic transmission (Pisu et al., 2011b; Talani et al., 2016; Lander et al., 2017; Wang et al., 2019), reduced PFC myelin-related gene (Makinodan et al., 2012), and protein expression (Hinton et al., 2019) and impaired plasticity (Quan et al., 2010; Medendorp et al., 2018; Wang et al., 2019). Isolation stress also leads to altered mesolimbic DA signaling including enhanced presynaptic DA and 5-HT function in the nucleus accumbens (Yorgason et al., 2013, 2016; Karkhanis et al., 2014), deficits in DA and 5-HT function in the PFC (Bibancos et al., 2007), and widespread changes across the brain with an altered structural connectome (Liu et al., 2016). Similarly, adolescent binge ethanol also induces structural changes, including reduction of myelin content in the frontal cortex (Vargas et al., 2014), myelin gene expression (Montesinos et al., 2014; Wolstenholme et al., 2017), and disrupts DA signaling (Coleman, He et al., 2011; Karkhanis et al., 2014; Trantham-Davidson et al., 2017). These effects appear specific to adolescents – when binge ethanol was administered to adult DBA/2J mice, myelin-related gene expression remained unaltered, indicating that adolescents are particularly susceptible to binge ethanol (Bent et al., 2022). A lack of social experience or exposure to ethanol during this critical brain developmental period likely alters the necessary stimulation from cortical brain regions, and, by impacting brain structure, molecular function, and neural connections, gives rise to impaired development and behavior that can persist into adulthood (Figure 1).

### Social Isolation or Binge Ethanol in Adolescence Negatively Impacts the Developing Glucocorticoid System

The glucocorticoid (GC) system undergoes development throughout adolescence. As there is a high density of GC receptors in the hippocampus, medial PFC, and amygdala (McCormick and Mathews, 2010), this system is particularly vulnerable to lasting effects of stress, including social stress. In adolescence, circulating corticosterone (CORT), the rodent analog to human cortisol, increases and then levels off in adulthood (Goldsmith et al., 1978; Meaney et al., 1985; Laviola et al., 2002; Silveri and Spear, 2004; Gunnar et al., 2009a; Stroud et al., 2009). Temporarily high CORT concentrations occupy both low-affinity GC receptors and high-affinity mineralocorticoid receptors to coordinate high rates of cortical dendritic spine turnover and pruning during adolescence (Liston and Gan, 2011). While the number of GC receptors in the brain does not differ between adolescents and adults, adolescents do not tend to habituate (measured as a blunted CORT release) to repeated stressors as compared to adults (Vazquez, 1998; Romeo et al., 2006). Single housing in adolescent rodents disrupts hypothalamus-pituitary axis (HPA) function (Butler et al., 2014a; Hinton et al., 2019), changes corticosterone response (Weiss et al., 2004), increases locomotor (Silva-Gomez et al., 2003; Voikar et al., 2005; Bianchi et al., 2006; Bibancos et al., 2007; Varlinskaya and Spear, 2008; McLean et al., 2010; Chappell et al., 2013; Butler et al., 2014a; Skelly et al., 2015; Lander et al., 2017; Medendorp et al., 2018; Wang et al., 2019) and anxiety-like behaviors (Weiss et al., 2004; Voikar et al., 2005; Koike et al., 2009; McCool and Chappell, 2009; Veenema, 2009; Makinodan et al., 2012; Chappell et al., 2013; Yorgason et al., 2013; Skelly et al., 2015; Lander et al., 2017). Social isolation in adolescent mice also leads to glucocorticoid insufficiency, increased density of dendritic spines in the PFC, and increased PSD-95 immunostaining suggesting that social isolation leads to improper synaptic pruning and retainment of overabundant dendritic spines (Hinton et al., 2019). Notably, adolescent ethanol-exposed mice show similar alterations in HPA function and anxiety-like behavior (Varlinskaya et al., 2015). The CORT response in adults with a history of adolescent binge ethanol (PND 25–45, 3.5 g/kg, i.g.), did not habituate to repeated restraint stress as would occur in ethanol naïve adults, suggesting a dysregulation of the HPA axis (Varlinskaya et al., 2016). Thus, it is likely that social isolation or binge ethanol in adolescence delays or prevents the age-appropriate maturation of the HPA axis. The ongoing refinement of circuitry between brain regions (i.e., PFC, nucleus accumbens, hippocampus, and amygdala) and the HPA axis, as well as the ongoing development of related signaling systems such as 5-HT, DA and GC, may help explain the biological reasons that social isolation or exposure to binge ethanol is particularly damaging to the development of an adolescent. Social isolation stress and/or ethanol exposure during this time disrupts the trajectory of cortical development and can lead to behavioral problems later in life, including attention, social, and cognitive deficits. Below, we review the areas of convergence and divergence in social isolation and binge drinking on social, ethanol-related, anxiety-like, and cognitive behaviors in rodent models (Tables 1, 2). We also discuss structural changes in brain regions important for these behaviors. We focus specifically on rodent studies using single housing as the method of social stress as these studies are widely used to model social isolation stress. Additionally, our review is largely confined to studies using primarily adolescent (∼PND 28–50) ethanol exposure.

**TABLE 1:** Behavioral, structural, and molecular impacts following adolescent social isolation.

Study	Strain (sex)	Isolation model (age of isolation onset)	Behavioral impacts (age of testing)	Structural impacts	Molecular impacts
Liu et al., 2019	CD-1 mice (M)	GH (5/cage) or SI (PND 23)	↑Soc affiliation, ↓soc rec, ↓soc memory (∼PND 93)		
Zhang et al., 2021	C57BL/6J mice (M)	GH (3/cage) or SI (PND 28)	↓Soc pref, ↓soc rec (5-trial soc rec task, ∼PND 56)		
Liu et al., 2015	C57BL/6J mice (M)	GH (3–5/cage) or (PND 21–22)	↓Soc interaction (∼4 weeks), ↑retention of fear memory (∼8 weeks)		
Kercmar et al., 2011	C57BL/6J mice (M&F)	GH (3/cage) or SI (PND 30–80 or PND 30–60)	↓Soc rec, (PND 80)		
Varlinskaya and Spear, 2008	Sprague–Dawley rats (M&F)	GH (3/cage) or SI from PND 23–28, PND 30–35, PND 37–42, or PND 65–70	Increased soc activity in familiar and unfamiliar testing situations at PND 28, increased soc activity only in unfamiliar testing situation at PND 35 and 42		
Skelly et al., 2015	Long-Evans rats (M)	GH (4/cage) or SI (PND 28)	↑Locomotion (OF, ∼PND 70), ↑anxiety-like behavior (OF, ∼PND 70), ↓fear extinction (∼PND 84), ↑intake (i-2BC, 20% EtOH)		
Naert et al., 2011	C57BL/6J mice (M)	GH (3/cage) or SI (PND 21)	↑Locomotion (OF, ∼PND 70), delayed fear extinction (∼PND 175)		
Bianchi et al., 2006	Lister hooded rats (M)	GH (3–4/cage) or SI (PND 25-28)	↑Locomotion (OF, PND 53–56), ↓NOR (PND 54–57)		
Voikar et al., 2005	C57BL/6J and DBA/2 mice (M)	GH (3/cage) or SI (PND 28)	↑Locomotion (OF), ↑anxiety-like behavior (EPM), ↓NOR, ↓freezing in contextual fear conditioning (PND 84∼112)		
McLean et al., 2010	Lister hooded rats (F)	GH (5/cage) or SI (PND 21-23)	↑Locomotion (OF), ↓NOR, only in 1 m and 1 h ITI, ↓set-shifting (∼9 weeks)		
McCool and Chappell, 2009	Long-Evans rats (M)	GH (4/cage) or SI (PND 28–32)	↑Anxiety-like behavior (EPM, ∼10 weeks), ↑intake and pref (24 h-access 2BC, 5 days, 10% EtOH), ↑operant response rate and intake (2 weeks, 10% EtOH)		
Chappell et al., 2013	Long-Evans rats (M)	GH (4-5/cage) or SI (PND 28–78 or PND 63–78)	↑Anxiety-like behavior (EPM, PND 72), ↑locomotion (OF, PND 74-78), ↑intake (24 h-access 2BC, 10% EtOH, PND 82–86 and i-2BC, 4 weeks, 20% EtOH, PND 89)		
Koike et al., 2009	ICR mice (M)	GH (5/cage) or SI (PND 21)	↑Anxiety-like behavior (EPM), ↓NOR, ↓habituation to unfamiliar intruder (∼4 weeks)		
Karkhanis et al., 2014	Long-Evans rats (M)	GH (4/cage) or SI (PND 28)	↑Anxiety-like behavior (EPM, PND 74–75)		↑DA and NE response with acute EtOH (2 g/kg, i.p.) in NAc
Yorgason et al., 2013	Long-Evans rats (M)	GH (4/cage) or SI (PND 28–77 or PND 28–174)	↑Anxiety-like behavior (EPM, PND 74 and PND 174)		↑DA terminal release and reuptake rates in NAc
Yorgason et al., 2016	Long-Evans rats (M)	GH (4/cage) or SI (PND 28–77 or PND 28–174)			↑DA terminal release and reuptake rates in NAc and dorsal medial striatum
Rivera-Irizarry et al., 2020	C57BL/6J mice (M&F)	GH (5/cage) or SI (PND 28 or PND 70)	↓Anxiety-like behavior (EPM, F only, PND 74), no diff in intake or pref (DID, 20–30% EtOH, PND 80–119), no memory deficits (NOR, PND 152), ↑soc interaction (PND 180)		
Lopez and Laber, 2015	C57BL/6J mice (M&F)	GH (4/cage) or SI ± enrichment (PND 21)	↑Intake in SI mice without enrichment (c-2BC, 3 weeks, 15% EtOH, PND 60), ↓anxiety-like behavior (LDB, PND 60)		
Lopez et al., 2011	C57BL/6J mice (M&F)	GH (4/cage) or SI (PND 21–61 or PND 60–100)	↑Intake (c-2BC, 2 weeks, 15% EtOH, PND 65 or PND 105)		
Butler et al., 2014b	Long-Evans rats (F)	GH (4/cage) or SI (PND 28)	↑Intake and pref at early timepoints (i-2BC, 7 weeks, 20% EtOH, PND 100)		
Deehan et al., 2007	Long-Evans rats (M)	GH (2/cage), enriched housing, or SI (PND 21)	↑Responding and pref (operant, 10% EtOH, PND 111)		
Advani et al., 2007	C57BL/6J mice (M&F)	Maternal separation (PND 2–14), then GH (2/cage) or SI (PND 21–60)	↑Intake in M; ↓intake in F, ↑pref (2BC Mon-Fri, 9 weeks, 5–20% EtOH, PND 60)		
Thorsell et al., 2005	Wistar rats (M)	GH (2/cage) or SI (PND 46)	No changes in intake (24 h access 2BC, 3 days, 10% EtOH, ∼13 weeks)		
Heimer-McGinn et al., 2020	Long-Evans rats (M)	GH (9/cage) or SI (PND 21)	↓Acquisition and reversal learning (BM, PND 143, 428, or 672)		
Ouchi et al., 2013	ICR mice (M)	GH (5/cage) or SI (PND 28–35), then resocialized for 5 weeks)	↓Contextual and conditional fear memory (∼9 weeks)		
Bagheri et al., 2021	NMRI mice (M)	GH (4/cage) or SI (PND 19–21)	↓Freezing behavior, ↓spatial memory (Y-maze, MWM, ∼8 weeks)		
Pais et al., 2019	C57BL/6J mice (M&F)	GH (4/cage) or SI (PND 29)	↓NOR (PND 63–65)		
Pisu et al., 2011a	Sprague-Dawley rats (M)	GH (6–8/cage) or SI (PND 25–30)	↑Spatial memory (MWM, PND 55–60)		↑ CORT on 5th day of MWM training
Lander et al., 2017	C57BL/6J mice (M)	GH or SI (PND 38 or PND 60)	↑Locomotion, ↑anxiety-like behavior (OF), ↑NOR, ↑soc pref, ↓reversal learning and set shifting (water T-maze, ∼PND 61 or 81)		↑Glutamatergic tone in mPFC
Wang et al., 2019	Balb/c mice (M)	GH or SI (PND 21)	↑Locomotion (OF), ↑swim speeds, ↓spatial memory (MWM, ∼28 weeks)	↓plasticity through ↓LTP in hippocampus (∼28 weeks)	↓ AMPAR, NMDAR, and PSD-95 protein in hippocampus (∼28 weeks)
Bibancos et al., 2007	C57BL/6J mice (M)	GH or SI (PND 21)	↑Locomotion (OF, EPM, LDB,∼6 weeks)		↓5-HT receptor mRNA in PFC (∼6 weeks)
Makinodan et al., 2012	PLP-eGFP mice (M)	GH (4/cage), enriched-housing (8/cage) or SI (PND 21)	Memory deficits (non-matching-to-place task), ↓ soc interaction, no diff in locomotion (PND 50)		↓PFC myelin-related mRNA (PND 65)
Butler et al., 2014a	Long-Evans rats (M)	GH (4/cage) or SI (PND 28)	↑Locomotion (OF, PND 78–79), ↑intake and pref (i-2BC, 6 weeks, 20% EtOH, PND 92–95)		Altered HPA axis function (PND 85–88)
Weiss et al., 2004	Sprague–Dawley rats (M&F)	GH or SI (PND 21)	↑Anxiety-like behavior (EPM, M only), ↓contextual fear conditioning (∼16 weeks)		Changes to CORT response (M only, ∼16 weeks)
Hinton et al., 2019	*Thy-1-YFP* C57BL/6J mice (M&F)	GH (6–8/cage) or SI (PND 31)		↑spine densities and PSD-95ir in OFC (PND 82)	GC insufficiency and ↓CNPase in cortico-striatal regions (PND 39)
Quan et al., 2010	Wistar rats (M)	GH (3–4/cage) or SI (PND 21)	↓Spatial memory and reversal learning (MWM, ∼8 weeks)	↓plasticity through ↓LTP in PFC (∼8 weeks)	
Liu et al., 2016	C57BL/6 mice (M&F)	GH (3–4/cage) or SI (PND 35)	↑Locomotion (OF), ↓freezing in contextual and tone fear conditioning (PND 57–61)	Altered structural connectivity (PND 57–61)	
Medendorp et al., 2018	Thy1-GFP mice (M&F)	GH or SI (PND 21)	↓Sociability (soc pref), ↑locomotion (OF, ∼PND 112)	Immature dendritic spines, unaltered spine density in mPFC, impaired plasticity through ↓LTP (∼PND 112)	
Cinini et al., 2014	Marmoset non-human primates (M&F)	GH with family for 3 weeks, or SI for 1 or 3 weeks at 8–10 months of age		↓ BrdU + cells in hippocampus, and a smaller percentage of these cells were co-labeled with DCX (8–10 months)	
Lesscher et al., 2015	Lister hooded rats (M)	GH (4/cage) or SI (PND 21–43, then 4 weeks of pair housing)	↑Intake and pref at early timepoints (i-2BC, 6 weeks period after 6 weeks of re-socialization, 20% EtOH)		
Han et al., 2011	Sprague Dawley rats (M)	GH (3/cage) or SI (PND 21–34, then resocialized from 35 to 55)	↓Reversal learning (MWM, ∼PND 55)		
Ouchi et al., 2013	ICR mice (M)	GH (5/cage) or SI (PND 28–35), then resocialized for 5 weeks)	↓Contextual and conditional fear memory (∼9 weeks)		
Biggio et al., 2019	Sprague-Dawley rats (M)	GH (5/cage), SI (PND 21) or SI + re-socialized (PND 49–77)		↓BrdU+ and Ki67 + cells (PND 78), ↓DCX + cells (PND 92), ↓spine density and branching, ↓mature, mushroom spines in DG (PND 77)	
Li et al., 2019	Sprague-Dawley rats (M)	GH (4/cage) or SI (PND 21–34, then resocialization from PND 35–55)	↓Reversal learning (MWM, PND 55)		
Talani et al., 2016	C57BL/6J mice (M)	GH (4–6/cage) or SI (PND 21)		↓neuronal excitability in DG	
Silva-Gomez et al., 2003	Sprague-Dawley rats (M)	GH (3/cage) or SI (PND 21–77)	↑Locomotion (OF, PND 70)	↓ proximal and distal spine density in mPFC layer III and hippocampal CA1; shorter dendritic length in CA1 only	

*Drinking data presented as (drinking paradigm, length of drinking period, percentage EtOH, PND drinking began) unless specific PNDs were given for drinking period. GH, group-housed; SI, socially isolated; PND, postnatal day; M, males; F, females; soc, social; rec, recognition; pref, preference; EPM, elevated plus maze; LDB, light-dark box; OF, open field; BM, Barnes maze; MWM, Morris water maze; NOR, novel object recognition; ITI, inter-trial interval; i.p., intraperitoneal; i.g., intragastric; i-2BC, intermittent two-bottle choice; c-2BC, consecutive 2-bottle choice; DID, drinking in the dark; PFC, prefrontal cortex; mPFC, medial prefrontal cortex; OFC, orbitofrontal cortex; NAc, nucleus accumbens; DG, dentate gyrus; GC, glucocorticoid; CORT, corticosterone; HPA, hypothalamic-pituitary-adrenal axis; DA, dopamine; 5-HT, serotonin; NE, norepinephrine; LTP, long-term potentiation.*

**TABLE 2:** Behavioral, structural, and molecular impacts following adolescent ethanol exposure.

Study	Strain (sex)	Ethanol paradigm (age of exposure)	Behavioral impacts	Structural impacts	Molecular impacts
**Acute ethanol exposure**					
Varlinskaya and Spear, 2002	Sprague Dawley rats (M&F)	Acute i.p., 0.25–4 g/kg, 12.6% EtOH, prior to testing (PND 35 or PND 70)	↑Sensitivity to EtOH-induced social facilitation and ↓sensitivity to EtOH-suppression of social interactions (PND 35 > PND 70)		
Varlinskaya and Spear, 2004	Sprague Dawley rats (M&F)	Acute i.p., 0.25–1.75 g/kg, 12.6% EtOH, prior to testing (PND 28, 35, or 42)	↑sensitivity to EtOH-induced social facilitation and ↓sensitivity to EtOH-suppression of social interactions (PND 28 > 35 and 42)		
Raymond et al., 2019	C57BL/6J mice (M)	Acute i.p., 0.25–1.6 g/kg, 3–19% EtOH (PND 31–33 or 10-weeks)	0.25 g/kg EtOH alleviated social avoidance and no social suppression at higher EtOH		
Lopez et al., 2003	C57BL/6J mice (M)	Acute i.p. prior to task, 1.0 g/kg, 1.75 g/kg, or 2.5 g/kg EtOH (PND 34–35 or PND 70)	↑Locomotion in adolescents (OF)		
Hefner and Holmes, 2007	C57BL/6J mice (M)	Acute i.p. prior to task, 1.5 g/kg (4, 6, or 8 weeks of age)	↑Locomotion in adolescents (OF)		
Stevenson et al., 2008	DBA/2J mice (M)	Acute i.p. prior to task, 1.5–3 g/kg, 20% EtOH (PND 28 or PND 63)	↑Locomotion in adolescents (OF)		
Melon and Boehm, 2011	C57BL/6J and DBA/2J mice (M&F)	Acute i.p., 2 g/kg, 20% EtOH (PND 28–32 or PND 60–80)	↑Locomotion in adolescents (OF)		
**Subchronic ethanol exposure**					
Coleman, He et al., 2011	C57BL/6 mice (M)	Consecutive i.g., 5 g/kg, 25% EtOH (PND 28–37 or PND 88–97)	↓Reversal learning (MWM, PND 63–72)		Decreased cholinergic and DA-related mRNA in whole brain (PND 38)
Quoilin et al., 2014	Swiss mice (F)	Consecutive i.p., 2.5 or 4 g/kg, 20% EtOH (PND 28–41 or PND 63–76)	↑Locomotion (OF, acute i.p., 2.5 g/kg, 20% EtOH, PND 63)		
Quoilin et al., 2012	Swiss mice (F)	Consecutive i.p., 2.5 or 4 g/kg, 20% EtOH (PND 28–41)	↑Locomotion (OF, acute i.p., 2.5 g/kg, 20% EtOH, PND 63)		
Sircar and Sircar, 2005	Sprague Dawley rats (M)	Consecutive i.p., 2 g/kg (PND 30–35 or PND 60–65)	↓Spatial memory (MWM, 30 min after last dose, PND 35) and when retested (PND 44, 48, and 60)		
Sircar et al., 2009	Sprague Dawley rats (F)	Consecutive i.p., 2 g/kg (PND 30–35 or PND 60–65)	↓Spatial memory (MWM, 30 min after last dose, PND 35)		
Carrara-Nascimento et al., 2017	Swiss mice (M)	Consecutive i.p., 2 g/kg, 20% EtOH (PND 30–45 or PND 70–85)	↑Adult intake (3BC, DID, periods of withdrawals and re-exposures, 4–15% EtOH, PND 50–136)		
Broadwater and Spear, 2013	Sprague Dawley rats (M)	Intermittent i.g., 4 g/kg, 25% EtOH (PND 28–48 or PND 70–90)	↓Retention in contextual fear conditioning in adulthood after early EtOH exposure		
Varlinskaya et al., 2014	Sprague Dawley rats (M&F)	Intermittent i.g., 3.5 g/kg, 25% EtOH (PND 25–45 or P45–P65)	↓Social investigation and pref (M only, PND 70), EtOH-induced social facilitation (acute i.p., 0.5–1.0 g/kg, 12.6% EtOH, PND 70)		
Varlinskaya et al., 2016	Sprague Dawley rats (M&F)	Intermittent i.g., 3.5 g/kg, 25% EtOH (PND 25–45)	↓Soc investigation and ↓soc pref (M only, PND 70 and 77)		Dysregulation of the HPA axis (no habituation to repeated restraint stress)
Scheidt et al., 2015	Wistar rats (M)	Intermittent i.g., 3 g/kg of 15% EtOH or 1 g/kg of 5% EtOH (PND 30–46)	No anxiety-like behavior (EPM, PND 64–65)		
Broadwater et al., 2014	Sprague Dawley rats (M)	Intermittent i.g., 4 g/kg (PND 28–48 or PND 70–90)		↓DCX + cells in DG (PND 74)	
Galaj et al., 2020	Sprague Dawley rats (M)	Intermittent i.g., 4 g/kg, 25% EtOH (PND 28–45 or PND 70–88)		↑Neuronal excitability and ↓spine density in prelimbic layer V (PND 109)	
Wolstenholme et al., 2017	DBA/2J mice (M&F)	Intermittent i.g., 4 g/kg, 25% EtOH (PND 29–42)	↑Locomotion (OF, PND 43), ↑locomotion in F after acute EtOH (OF, PND 66), ↓NOR (PND 66+)	↓Myelin-related mRNA (PND 43)	
Vetreno and Crews, 2015	Wistar rats (M)	Intermittent i.g., 5 g/kg, 20% EtOH (PND 25–55)	↓NOR (PND 163–165)	Persistently ↓ DCX+ and Ki67+ cells in dorsal and ventral hippocampus (PND 56–220)	
Vetreno and Crews, 2012	Wistar rats (M)	Intermittent i.g., 5 g/kg, 20% EtOH (PND 25–55)	↓Reversal learning (MWM, PND 70)		
Vetreno et al., 2016	Wistar rats (M)	Intermittent i.g., 5 g/kg, 20% EtOH (PND 25–55)	↓NOR (PND 163–165), ↑anxiety-like behavior (LDB, PND 219)		
Kipp et al., 2021	Sprague Dawley rats (M&F)	Intermittent i.g., 5 g/kg, 20% EtOH (PND 25–55)	No spatial memory deficits (SA, EPM), No deficits in reversal learning (operant model, PND 55+)	↑Branching of apical dendric trees in the OFC (PND 115–125)	
Liu and Crews, 2017	Wistar rats (M)	Intermittent i.g., 5 g/kg, 25% EtOH (PND 25–54)		↓BrdU+, Ki67+, DCX+, Sox2+, and Tbr2+ cells in DG (PND 95)	
Coleman, Liu et al., 2014	C57BL/6J mice (M&F)	Intermittent i.g., 5 g/kg, 25% EtOH (PND 28–37)	↓Reversal learning (BM, PND 91)		
Risher et al., 2015	Sprague Dawley rats (M)	Intermittent i.g., 5 g/kg, 35% EtOH (PND 30–46)		↑Density of immature and ↓density of mature spines and ↑likelihood of low stimulus LTP in CA1 (PND 70–75)	
Mulholland et al., 2018	Sprague Dawley rats (M)	Intermittent i.g., 5 g/kg, 35% EtOH (PND 30–46)		↓Density of long and mushroom spines ↓volume and diameter of long and stubby spines and filopodia in DG (PND 70)	
Pandey et al., 2015	Sprague Dawley rats (M)	Intermittent i.p., 2 g/kg, 20% EtOH (PND 28–41)	↑Anxiety-like behavior (LDB, EPM, PND 42)		
Sakharkar et al., 2016	Sprague Dawley rats (M)	Intermittent i.p., 2g/kg, 20% EtOH (PND 28-41)	↑Anxiety-like behavior (LDB, PND 94)	↓DCX+ and Ki67+ cells in DG (PND 92)	
Pascual et al., 2007	Wistar rats (M)	Intermittent i.p., 3 g/kg, 25% EtOH (PND 25–38)	↓NOR (PND 41 and 61)		
Montesinos et al., 2014	C57BL/6 mice (F)	Intermittent i.p., 3 g/kg, 25% EtOH (PND 30–43)	↓NOR (PND 66+)	↓Myelin-related mRNA (PND 44), altered myelin structure in PFC (PND 44 and 65)	
Montesinos et al., 2016	C57BL/6 mice (F)	Intermittent i.p., 3 g/kg, 25% EtOH (PND 30–43)	↑Adult pref (PND 66+), ↑anxiety-like behavior (OF, EPM, PND 66+)		
Pascual et al., 2021	C57BL/6J mice (M&F)	Intermittent i.p., 3 g/kg, 25% EtOH (PND 30–43)	↓NOR (PND 46)	↑Spine density in DG (thin spines in F and stubby spines in M, PND 44)	
**Drinking in the Dark**					
Szumlinski et al., 2019	C57BL/6J mice (M&F)	2, 3, or 4 BC, DID, 5–40% EtOH for 14 days (PND 28–29 or PND 56–58)	↑Anxiety-like behavior (LDB, PND ∼72)		
Lee et al., 2018	C57BL/6J mice (M)	3BC, DID, 10–40% EtOH (PND 28–42 or PND 56–70)	↑Adult intake (3BC, DID, 10–40% EtOH, PND ∼71–76), ↑anxiety-like behavior (LDB, PND 70)		
Lee et al., 2016	C57BL/6J mice (M)	4BC, DID, 5–40% EtOH (4–6 weeks or 8–10 weeks)	No memory deficits (NOR, 24 h after last drink), no anxiety-like behavior (marble burying, 48 h after last drink)		
Lee et al., 2017	C57BL/6J mice (M)	4BC, DID, 5–40% EtOH (PND 28–42 or PND 56–70)	↑Anxiety-like behavior (marble burying, PND 70), ↑adult intake (4BC, DID, 5–40% EtOH, PND ∼71–76)		
Metten et al., 2011	HS/Np or HDID-1 mice (M&F)	DID, 2-week period, 20% EtOH (3–4, 4–5, 5–6, 6–7, 7–8, or 8–9 weeks)	↑Adult intake (DID, 4 days on, 3 days off, 2-week period, 4-15% EtOH, 9 weeks)		
Younis et al., 2019	C57BL/6J mice (M&F)	DID, 20% EtOH (PND 28–36 or PND 72–80)	↑Adult intake and pref (DID, 20% EtOH and c-2BC, 5–15% EtOH, PND 72–80)		
Moore et al., 2010	C57BL/6J and DBA/2J mice (M&F)	DID, 20% EtOH (PND 28–42)	↑Adult intake (C57B/6J only, DID, 20% EtOH, PND 63–77)		
Marco et al., 2017	Wistar rats (M&F)	DID, 20% EtOH (PND 28–52)	No anxiety-like behavior (EPM, PND 53), ↓NOR (PND 63)		
Wolstenholme et al., 2020	C57BL/6J and DBA/2J mice (M)	DID, 5 or 20% EtOH (PND 28–36)	↑Adult intake (DID, 5% EtOH, PND 72–80)		
**2-bottle choice drinking**					
Lesscher et al., 2021	Lister hooded rats (M)	i-2BC, 20% EtOH (PND 42-56)	↑Adult intake (i-2BC, 8 weeks, 20% EtOH, PND 75)		
Salling et al., 2018	C57BL/6J mice (M)	i-2BC, 15% EtOH (∼PND 30–60)	No anxiety-like behavior (EPM, ∼PND 63), ↓memory (delayed non-match to sample in T-maze, PND 65–80), No increase in adult intake (i-2BC, 15% EtOH, PND 70–94)		
Varlinskaya et al., 2015	Sprague Dawley rats (M&F)	i-2BC, 10% EtOH, access occurred alone or with 4–5 littermates (PND 36–46 or PND 76–86)	↓Intake in F when drinking alone, ↑intake in M when social drinking		
**Vapor exposure**					
Jury et al., 2017	C57BL/6 or Thy-1 mice (M)	CIE (4–6 or 8–10 weeks)	No changes in intake or pref (24-h access 2BC, 2-week period, 15% EtOH, ∼10 weeks)	↓Spine density in infralimbic mPFC, adult mice had thinner thin spines and wider wide spines	
Gass et al., 2014	Long–Evans rats (M)	Intermittent vapor exposure (PND 28–42)	↑Self-admin (operant model, 10–20% EtOH, PND 65–90), ↓cognitive flexibility (set shifting, PND 90–130), ↓anxiety-like behavior (EPM, PND 90–130), ↑resistance to extinction of EtOH-seeking (PND 90–130)		
Trantham-Davidson et al., 2017	Long–Evans rats (M)	Intermittent vapor exposure (PND 28–42)		↑Long thin spine density in layer V of the prelimbic mPFC in adulthood	Alteration of DA neurotransmission in the prelimbic mPFC in adulthood
Amodeo et al., 2021	Wistar rats (M)	Intermittent CIE, 95% EtOH (PND 22–57 or PND 91–126)		↓Long spine density in primary motor cortex (PND 70), ↓immature spine density in primary visual cortex at all ages (PND 70 and PND 139)	
**Other**					
Vargas et al., 2014	Wistar rats (M)	Operant model, 8–10% sweetened EtOH (PND 28–42 and PND 78–130)	↓Working memory (T-maze, PND 88–89)	↓Myelin density in mPFC (PND 43)	
Strong et al., 2010	C57BL/6J mice (M&F)	Scheduled high alcohol consumption, 21 days access (PND 26–28 or PND 58–71)	↑Adult pref (c-2BC, 20% EtOH, PND 66–69 and 24 h access 2BC, 5% EtOH, PND 58–61), ↑adult intake [24 h access 2BC, 5% EtOH, (M&F) and 10% EtOH (F only), PND 58–61]		

*Drinking data presented as (drinking paradigm, length of drinking period, percentage EtOH, PND drinking began) unless specific PNDs were given for drinking period. PND, postnatal day; M, males; F, females; i.p., intraperitoneal; i.g., intragastric; BC, bottle choice, i-2BC, intermittent two-bottle choice; c-2BC, consecutive 2-bottle choice; DID, drinking in the dark; soc, social; pref, preference; EPM, elevated plus maze; LDB, light-dark box; OF, open field; SA, spontaneous alternation; BM, Barnes maze; MWM, Morris water maze; NOR, novel object recognition; ITI, inter-trial interval; PFC, prefrontal cortex; mPFC, medial prefrontal cortex; OFC, orbitofrontal cortex; DG, dentate gyrus; HPA, hypothalamic-pituitary-adrenal axis; DA, dopamine; LTP, long-term potentiation.*

### Social Isolation Increases Social Behavior and Disrupts Social Memory, While Ethanol Elicits Dose-Dependent Effects on Social Behavior

There is an age-dependent proclivity toward social interaction in both humans and rodents, with adolescents interacting more with a novel conspecific than adults. Adolescent social isolation tends to increase social interactions in adolescence and in adulthood (Table 1). Five days of social isolation in adolescent rats increased pro-social behavior in an age-dependent manner as compared to group housed rats (Varlinskaya and Spear, 2008). Single-housed rats displayed increases in social investigation frequency, contact behavior, and play fighting behavior compared to group housed rats and this effect was greatest in young adolescent (PND 28) rats (Varlinskaya and Spear, 2008). Socially isolated male and female C57BL/6J mice also showed increased social interaction following isolation from PND 28–70 (Rivera-Irizarry et al., 2020). Conversely, group housed C57BL/6 (Liu et al., 2015) mice or PLP-eGFP mice (Makinodan et al., 2012) were more interactive with a novel conspecific than socially isolated mice.

Social recognition, the ability to remember and discriminate a previously encountered conspecific, is an important component of social behavior. The multi-trial social recognition task and the 3-chamber social preference task are frequently used to assess social memory and social approach in rodents. The 3-chamber social preference task consists of two phases to measure social approach (preference for an empty chamber versus a chamber with a novel stimulus mouse) and social memory (preference for a novel versus familiar stimulus). In the 3-chamber social preference task, Thy1-GFP males and females (Medendorp et al., 2018) and socially isolated C56BL/6 males (Zhang et al., 2021) did not differ in their social affiliation as compared to group housed males and displayed a typical preference for a stimulus animal over an empty cylinder. Thy1-GFP mice are on a C57BL/6 genetic background and were used to allow for imaging of pyramidal neurons. Socially isolated CD1 males, however, spent more time with the stimulus mouse during both phases of the test (Liu et al., 2019). During the second phase to assess social memory, adolescent social isolation decreased the interaction time with novel mouse over the familiar, suggesting poor social memory in both male and female Thy1-GFP mice (Medendorp et al., 2018), C57BL/6 males (Zhang et al., 2021), and CD1 males (Liu et al., 2019). Similarly, in a 5 or 9-trial social recognition task, postweaning social isolation in CD1 males and C57BL/6 males and females increased social interactions to the stimulus mouse during the habituation phases and decreased their ability to recognize a novel mouse during the dishabituation phase, suggesting a deficit in social memory (Kercmar et al., 2011; Liu et al., 2019; Zhang et al., 2021). In a modified social interaction task, socially isolated males retained higher frequencies of social interaction and did not habituate to repeated exposures to the same stimulus mouse; mice were not tested for the dishabituation phase (Koike et al., 2009). Together, when tested for social interaction, social play, or in social memory-related tasks, adolescent social isolation in rodents typically increases overall social interactions and this increased interaction may preclude the ability to form a memory of the novel animal.

Most studies investigating ethanol effects on sociability have measured social behavior with acute ethanol administration, while ethanol was still present in their system (Table 2). In response to acute low doses of ethanol, juveniles show social facilitation and are more sensitive to ethanol’s rewarding effects than adults, increasing the incentive value for adolescents (Varlinskaya et al., 2014). In rats, low ethanol doses (0.25–0.75 g/kg) increased social activity and social preference for a novel rat, while high doses (3–4 g/kg) decreased social activity and increased peer avoidance (Varlinskaya and Spear, 2002). Adolescent rats are more sensitive to social facilitation and are less sensitive to the suppression of social interactions compared to adults; early adolescents (PND 28) show even more social facilitation than mid to late adolescents (PND 35 and 42) (Varlinskaya and Spear, 2004). Similarly in mice, acute high doses of ethanol (1.6 g/kg) impaired social approach in adult mice, while low doses (0.25 g/kg) alleviated social anxiety-like avoidance and high doses did not suppress social interactions in adolescents (Raymond et al., 2019). Adult male rats with a history of ethanol (PND 28–42, 3.5 g/kg, i.g., every other day) showed enhanced sensitivity to the social-facilitating effects of ethanol (Varlinskaya et al., 2014). Adult males with a history of ethanol were also more social with a conspecific than ethanol-naïve males, suggesting that the males retained an adolescent-typical responsiveness to alcohol; females were insensitive to the social modulating effects of ethanol (Varlinskaya et al., 2014). Ethanol’s effects on social memory in the social recognition task or 3-chamber social preference task have not been investigated.

### Adolescents Consume More Ethanol Than Adults and Social Experience Modifies Ethanol Consumption

Adolescents are less sensitive than adults to the social impairing, motor disrupting, aversive, and sedative effects of higher doses of ethanol (Varlinskaya and Spear, 2002; Varlinskaya et al., 2016), allowing adolescents to consume more ethanol than their adult counterparts (Ho et al., 1989; Tambour et al., 2008; Maldonado-Devincci et al., 2010; Strong et al., 2010; Metten et al., 2011). Prior exposure to ethanol tends to increase drinking later in life and this effect is stronger when binge ethanol exposure occurs during adolescence. Early life binge drinking, but not adult binge drinking, increases adult ethanol intake and preference (Moore et al., 2010; Strong et al., 2010; Metten et al., 2011; Montesinos et al., 2016; Carrara-Nascimento et al., 2017; Lee et al., 2017; Younis et al., 2019; Wolstenholme et al., 2020; Lesscher et al., 2021). As compared to animals that first received ethanol in adulthood, adolescent ethanol exposure tends to increase later voluntary consumption in adulthood (Younis et al., 2019; Lesscher et al., 2021). In a set of studies to determine the optimal age for adolescent ethanol exposure to increase adult intake, Metten et al. (2011) tested six ages of HS/Npt male mice, a genetically heterogeneous mouse strain, and found that ethanol exposure beginning in mid-adolescence (PND 28) is most effective at increasing ethanol intake in adulthood (Metten et al., 2011). Similarly, mice selectively bred for high ethanol consumption, HDID-1 mice, first exposed to ethanol DID at 4 weeks, consumed significantly more ethanol than HDID-1 mice first exposed to ethanol at 6 or 9 weeks of age (Metten et al., 2011). In C57BL/6J males, DID from PND 28–36 increased adult DID as well as adult ethanol intake and preference in the 2-bottle choice model (2BC), whereas DID exposure in adults (from PND 73-81) did not alter ethanol intake or preference 36 days later (Younis et al., 2019). The number of ethanol exposures also plays a role in whether an animal increases ethanol consumption later in life. In C57BL/6J male mice, daily DID sessions from PND 28–36 increased adult intake, while intermittent, every other day DID sessions only moderately increased adult intake (Younis et al., 2019). In adult mice, repeated DID cycles during adulthood increased subsequent voluntary ethanol intake and preference, and this effect became more robust with increasing DID cycles (Cox et al., 2013), further suggesting that a minimum number of ethanol exposures may be needed to predispose animals to consume more ethanol upon re-exposure. However, adolescent exposure to ethanol does not always lead to increased intake in adulthood. Male Thy1-GFP mice given 2 weeks of 2BC ethanol on weeks 4–6 and then 4 cycles of chronic intermittent ethanol vapor (CIE) did not increase their ethanol intake or preference when tested again for 2BC drinking in adulthood (∼10 + weeks) (Jury et al., 2017). Adult mice exposed to 2BC drinking (8–10 weeks) and CIE increased their intake relative to air-exposed controls (Jury et al., 2017). This lack of increased intake in adolescents with a history of CIE may be due to a ceiling effect in mice on a C57BL/6 background, as suggested by the authors (Jury et al., 2017). Together, these studies indicate that age of exposure and perhaps a minimum number of ethanol exposures in adolescence, as well as genetic background, are all critical factors that influence the ability to detect a reliable increase adult drinking (Tables 2, 3).

**TABLE 3:** Behavioral, structural, and molecular impacts following combined adolescent social isolation and adolescent ethanol exposure.

Study	Strain (sex)	Isolation model (age of isolation onset)	EtOH paradigm	Behavioral impacts	Structural impacts	Molecular impacts
Sanna et al., 2011	C57BL/6J mice (M)	GH (6/cage) or SI (PND 21)	c-2BC, 15% EtOH (only for EtOH intake experiment)	↑Intake and pref due to SI, ↓locomotion due to SI (OF, 9 weeks)		↑α4 and δ GABA mRNA subunits in hippocampus due to SI (9 weeks)
Schenk et al., 1990	Long-Evans rats (M)	GH (4/cage) or SI (PND 21 or PND 65)	c-2BC, 10% EtOH	↑Intake and pref due to SI		
Pisu et al., 2011b	Sprague-Dawley rats (M)	GH (3/cage) or SI (PND 25)	24 h access 2BC, 4 weeks, 2.5–10% EtOH, PND 25–53	↓Pref due to SI, ↑anxiety-like behavior (EPM)		↑α4 (SI) and δ (SI and EtOH) GABA mRNA subunits in hippocampus

*Drinking data presented as (drinking paradigm, length of drinking period, percentage EtOH, PND drinking began) unless specific PNDs were given for drinking period. GH, group-housed; SI, social isolation; PND, postnatal day; M, males; F, females; i.p., intraperitoneal; i.g., intragastric; BC, bottle choice; c-2BC, consecutive 2-bottle choice; pref, preference; OF, open field; EPM, elevated plus maze.*

Using a variety of voluntary drinking models, adolescent social isolation without a history of adolescent drinking leads to increased drinking in adulthood in 2BC (Chappell et al., 2013; Butler et al., 2014a; Lesscher et al., 2015; Skelly et al., 2015), DID (Sanna et al., 2011), and operant responding models (Deehan et al., 2007; McCool and Chappell, 2009). In the few studies that have directly investigated drinking behavior in single versus group housed rodents, social isolation usually increases ethanol consumption behaviors in rats (Schenk et al., 1990; Wolffgramm, 1990; Chappell et al., 2013; Skelly et al., 2015) and mice (Advani et al., 2007; Lopez et al., 2011; Sanna et al., 2011). These effects appear to be limited to adolescence, as increased ethanol preference does not occur in isolated adult rodents (Schenk et al., 1990; Lopez et al., 2011; Chappell et al., 2013), suggesting that early timing of social isolation is crucial to produce significant changes in the sensitivity to ethanol in adulthood (Tables 1–3).

Not all studies have found similar effects of social housing on ethanol drinking. When tested in adolescence, single housed adolescent rats and group housed rats drank the same amounts, and single housed rats showed a decreased preference for ethanol in a 2BC continuous access model (Pisu et al., 2011b). In a 3-day 24 h access model, single and pair-housed rats consumed similar amounts of ethanol (Thorsell et al., 2005). Using a DID model, social isolation did not produce any differences in ethanol intake, preference, quinine-adulterated ethanol intake and preference, or sucrose preference in male or female mice (Rivera-Irizarry et al., 2020). Interestingly, high levels of social activity in adolescent male rats and high levels of social anxiety-like behavior in adolescent female rats were associated with elevated social drinking, suggesting that males ingest ethanol for its socially enhancing properties, while females may ingest ethanol for its social anxiolytic effects (Varlinskaya et al., 2015).

In general, female rodents, consume greater amounts of ethanol than their male counterparts (Vetter-O’Hagen et al., 2009; Lopez et al., 2011; Alfonso-Loeches et al., 2013; Lopez and Laber, 2015). A few studies have directly investigated sex differences in adult drinking following adolescent social isolation (Advani et al., 2007; Lopez et al., 2011; Butler et al., 2014a,b; Lopez and Laber, 2015; Rivera-Irizarry et al., 2020). Male and female mice with a history of social isolation in adolescence increased ethanol intake as compared to group housed mice, or mice isolated in adulthood, with effects stronger in males (Lopez et al., 2011; Lopez and Laber, 2015). When social isolation occurred in adult females, they consumed moderately less ethanol than group housed females (Lopez et al., 2011). Social isolation in adolescent male (Butler et al., 2014a) and female (Butler et al., 2014b) rats increased ethanol intake relative to group housed rats and effects were again stronger in males. In rats, single housed adolescent males and adult male and female rats consumed more sweetened alcohol under social circumstances than when individually housed, while single housed adolescent females consumed more ethanol than group housed females (Varlinskaya et al., 2015). Male rats exposed to ethanol during early adolescence retained an adolescent-like ethanol-induced social facilitation not normally seen in adults (Varlinskaya et al., 2014). Single housed male and female mice had increased ethanol preference during their first week of drinking at PND 60 which was maintained in adult males (Advani et al., 2007). But in adulthood, single housed females decreased their intake relative to pair housed mice (Advani et al., 2007). Socially isolated male rats showed robust increases in anxiety and ethanol intake compared to group housed males (Chappell et al., 2013; Skelly et al., 2015).

### Consequences of Adolescent Social Isolation and Binge Ethanol on Anxiety-Like Behaviors Are Mixed

The effects of adolescent social isolation on anxiety-like behaviors are mixed and may be confounded by hyperlocomotion (Table 1). Social isolation in adolescent rats increases anxiety-like behavior in the elevated plus maze (McCool and Chappell, 2009; Pisu et al., 2011b; Chappell et al., 2013; Yorgason et al., 2013; Skelly et al., 2015). In the open field task, overall locomotor activity was increased following social isolation in rats and mice (Bibancos et al., 2007; Varlinskaya and Spear, 2008; McLean et al., 2010; Skelly et al., 2015; Liu et al., 2016; Medendorp et al., 2018; Wang et al., 2019). Anxiety-dependent measures in the open field, such as time in the center, did not differ between housing groups (Medendorp et al., 2018; Wang et al., 2019). In male mice single housed in late adolescence (PND 38), time in center of the open field was lower, without a change in total locomotor activity (Lander et al., 2017). However, when tested directly for locomotor activity in a longer 30-min task, single housed mice were hyperactive (Lander et al., 2017). Time in the open arms was also lower in single housed ICR males, suggesting social isolation increased anxiety-like behavior (Koike et al., 2009). Elevated locomotor activity is not always found, as no differences in locomotor activity in a novel arena have also been reported between single and group housed animals (Koike et al., 2009; Liu et al., 2015; Hinton et al., 2019). Similarly, in the elevated plus maze task, no differences were found between adolescent single and group housed animals for time spent in the open arms (Hinton et al., 2019). Adolescent social isolation increased the latency to enter the light side of the light-dark box compared to group housed mice, but the time in the light did not differ (Pais et al., 2019).

Following bouts of binge ethanol, animals may display withdrawal symptoms and this negative affective state has been assessed using anxiety-like behavior tasks. Adult animals typically display increased anxiety-like behavior in the elevated plus maze or the light-dark box while in ethanol withdrawal (Lee et al., 2018; Szumlinski et al., 2019), potentially in an intake-dependent manner (Lee et al., 2015). Twenty-four hours after the last adolescent ethanol binge, rats only sometimes display withdrawal-induced anxiety phenotypes (Pandey et al., 2015; Kyzar et al., 2016), some of which persist long-term (Vetreno et al., 2016). However, similar to adolescent social isolation, binge ethanol in adolescence does not always affect anxiety-like behavior (Table 2). In adolescent mice and rats, withdrawal-induced increases in anxiety-like behavior were not observed in the light-dark box or the elevated plus maze (Scheidt et al., 2015; Lee et al., 2016, 2018; Marco et al., 2017; Wolstenholme et al., 2017; Salling et al., 2018). It is possible that these inconsistencies may be due to procedural differences, differences in route of administration, or that binge ethanol in adolescence only produces lasting withdrawal-induced anxiety in rats and not mice. One possibility, as some have suggested, is that adolescent mice are resilient to early ethanol withdrawal (Lee et al., 2016, 2018). A recent review of the literature in adult models of negative affective behavior during ethanol abstinence has highlighted similar highly variable findings and has promoted more data driven approaches are needed toward understanding this complex interaction (Bloch et al., 2022).

Overall, the results of social isolation or binge ethanol in adolescence have given inconsistent results on anxiety-like behavior. As discussed above, locomotor differences can confound the interpretation of anxiety-phenotypes as these tasks typically rely on locomotor activity. While testing for anxiety 24 h after the last binge, ethanol exposed DBA2/J mice traveled farther in the light–dark apparatus than controls (Wolstenholme et al., 2017). In adulthood, a priming injection of 2 g/kg ethanol increased locomotor activity in females with a history of binge ethanol while testing for ethanol-induced anxiolysis (Wolstenholme et al., 2017). Adolescent rodents are also more sensitive to low dose ethanol’s locomotor stimulating effects than adults (Lopez et al., 2003; Hefner and Holmes, 2007; Stevenson et al., 2008; Melon and Boehm, 2011). Swiss mice exposed to ethanol as adolescents, especially at high doses, have enhanced locomotor stimulant effects as compared to controls and these effects persisted for 3 weeks after the last ethanol exposure (Quoilin et al., 2012, 2014). It is possible that the general increased locomotor activity of socially isolated or ethanol exposed adolescents may overshadow the display of anxiety-like phenotypes in tasks that require locomotor activity. This heightened activity has the potential to confound the experimenter’s ability to interpret anxiety-like behaviors following social stress or exposure to binge ethanol. Alternatively, as adolescence may be a period of heightened vulnerability to the deleterious behavioral and neurobiological effects of ethanol or social isolation, these insults may increase stress reactivity (i.e., locomotor activity in a novel environment). Clearly, new approaches or methods for analyzing these complex data are needed to fully understand the interaction between social stress, ethanol and negative affect.

### Social Isolation and Binge Ethanol in Adolescence Lead to Deficits in Cognitive Behavior and Reversal Learning

There is a considerable amount of convergence in the effects of adolescent social isolation and binge ethanol on cognitive behaviors (Tables 1–3). Both adolescent social isolation and binge ethanol cause long-term deficits in recognition memory and cognitive flexibility. Relying on a rodents’ innate preference for novelty, the novel object recognition (NOR) test can be reliably used to assess both working and long-term memory. In this single trial task, an animal is allowed to explore a novel and a familiar object in a test arena. An intact short term or working memory is interpreted as higher preference for the novel object (see Antunes and Biala, 2012 for review). Adolescent social isolation decreased preference for the novel object in both rats and mice (Voikar et al., 2005; Bianchi et al., 2006; Koike et al., 2009; McLean et al., 2010; Pais et al., 2019). Following six weeks of social isolation, adult rats were able to discriminate between the original and novel objects in a 1 minute and 1 hour inter-trial interval (ITI), but not a 3.5 or 4 h ITI (McLean et al., 2010). Binge ethanol in adolescence also caused deficits in working or long-term memory when tested in adulthood (Pascual et al., 2007; Montesinos et al., 2014; Vetreno and Crews, 2015; Vetreno et al., 2016; Marco et al., 2017; Wolstenholme et al., 2017). Interestingly, binge ethanol in adolescence led to deficits in the NOR task primarily when mice were tested in adulthood and NOR deficits are not always observed in adolescence shortly after ethanol administration (Bent et al., 2022), suggesting a maturation of the brain circuitry in adulthood is necessary to observe these deficits. However, others have reported reduced performance in the NOR test in both late adolescent and adult rats after binge ethanol in adolescence (Pascual et al., 2007) and in late adolescent mice (Pascual et al., 2021). Of note, adult males and females that received the same binge paradigm as adolescent mice (4 g/kg, i.g.) did not display memory deficits in the NOR task (Bent et al., 2022). The combined effects of social isolation and binge ethanol have not been directly explored in the NOR task.

The Morris Water Maze (MWM) and Barnes Maze (BM) are two commonly used, multi-day tasks used to assess spatial memory and cognitive flexibility and acquisition of this task is largely hippocampal-dependent (Morris et al., 1982). The effects of social isolation beginning in adolescence are inconsistent for the acquisition of spatial learning. Long-term social isolation (6 months+) in Balb/c mice led to deficits in the acquisition and retention of spatial memory in the MWM, and this was accompanied by higher swim speeds (Wang et al., 2019), reminiscent of isolation-induced hyperactivity in the open field task. Five weeks of social isolation in male mice (Bagheri et al., 2021) also led to higher escape latencies and less time spent in the target quadrant in the MWM, when compared to socially housed mice. In the BM, 5 months of social isolation beginning at weaning impaired acquisition of both standard and reversal learning in male rats (Heimer-McGinn et al., 2020). In some studies, social isolation rearing did not impair (Quan et al., 2010; Han et al., 2011; Li et al., 2019) or improved spatial learning (Pisu et al., 2011a). In one study, adolescent-isolated adult rats had lower latencies to reach the MWM platform, and this was accompanied by a higher swim speed in the isolated animals and increased CORT levels on training day 5 (Pisu et al., 2011a), suggesting heightened anxiety-like or hyperactive behavior in socially isolated rats. During the reversal phase of the MWM to assess PFC-mediated cognitive flexibility, 2 weeks of social isolation in early adolescence led to reversal learning deficits in male rats, without housing effects on the spatial learning of the task (Li et al., 2019). Eight weeks of chronic isolation rearing in male rats did not affect the original acquisition learning, but impaired reversal learning in the MWM (Quan et al., 2010). Brief isolation in early adolescent male rats (isolated from PND 21–34 and re-socialized from PND 35–55) affected reversal learning without interfering with spatial learning in the MWM (Han et al., 2011). Attentional set-shifting was also impaired in isolation reared rats (McLean et al., 2010), and mice (Lander et al., 2017) further supporting PFC-related cognitive deficits from social isolation.

In most studies, animals with a history of binge ethanol are able to acquire spatial learning, suggesting that hippocampal-mediated spatial memory remains intact. Daily ethanol injections (2 g/kg, i.p.) immediately prior to testing in adolescent (PND 30) or adult (PND 60) male (Sircar and Sircar, 2005) and female (Sircar et al., 2009) rats impaired the acquisition of spatial reference memory in the MWM and the effects were stronger in adolescents. When tested up to 30 days after the last ethanol administration, ethanol-treated adolescents, but not adults, traveled longer distances to find the hidden platform than saline-treated controls (Sircar and Sircar, 2005), indicating that adolescent, but not adult, animals retained persistent deficits in spatial memory. However, in other studies, a history of ethanol exposure during adolescence did not lead to deficits in the spatial learning of the MWM (Coleman, He et al., 2011; Li et al., 2019) or the BM (Vetreno and Crews, 2012; Coleman, Liu et al., 2014). During the reversal phase, to test cognitive flexibility, adult male C57BL/6J mice with a history of adolescent ethanol (PND 28–37, 5 g/kg, i.g.) showed deficits in acquisition of a reversal Barnes maze (Coleman, Liu et al., 2014) and deficits in the reversal probe trial accompanied by a perseveration-like search strategy and more time spent in the standard MWM quadrant (Coleman, He et al., 2011). Rats exposed to CIE during adolescence (PND 28–42) were unable to alter their responses on a set-shifting task, indicative of impaired cognitive flexibility (Gass et al., 2014).

Similar to MWM and BM, acquisition of the fear conditioning task is not usually affected by adolescent social isolation, binge ethanol, or age. Contextual fear conditioning is primarily hippocampal dependent, whereas the mPFC (particularly the infralimbic cortex) is more associated with fear memory (Myers et al., 2006). Socially isolated rats continued to exhibit a fear-potentiated startle response and deficits in fear extinction learning, while the acquisition of contextual fear conditioning was unaffected by housing condition (Skelly et al., 2015). Isolation during early-mid adolescence produces deficits in extinction of contextual fear conditioning as far as 4 weeks after acquisition (Liu et al., 2015). Single housed mice eventually acquired extinction learning, but at a markedly slower rate than group housed conspecifics (Naert et al., 2011). Some studies report more hippocampal-mediated deficits in contextual fear conditioning 24 h after the initial training session following social isolation (Weiss et al., 2004; Voikar et al., 2005; Bagheri et al., 2021). Social isolation impaired both contextual and conditional fear memory in a fear conditioning test, indicative of impaired acquisition of the task (Ouchi et al., 2013; Liu et al., 2016). These changes could be mediated by altered structural connectivity among corticolimbic regions (Liu et al., 2016) or through the cholinergic system (Ouchi et al., 2013). Early adolescent exposure (PND 28–48) to ethanol impaired retention of the context in which animals had received a foot shock; late adolescent (PND 35–55) or adult (PND 70–90) exposure to ethanol resulted in impaired extinction of fear conditioning (Broadwater and Spear, 2013). Together, these data suggest that adolescent social isolation or binge ethanol leads to deficits in behavioral flexibility and increased perseveration of previously learned behaviors.

### Social Isolation and Binge Ethanol Alter Dendritic Morphology and Impair Neurogenesis

#### Dendritic Spine Density and Morphology

Dendritic spines are highly plastic and can be transiently modified by a single event or more permanently altered by chronic exposure to social isolation stress or binge ethanol (Silva-Gomez et al., 2003; Grossman et al., 2010; Trantham-Davidson et al., 2014; McGuier et al., 2015; Pandey et al., 2015; Risher et al., 2015; Jury et al., 2017; Medendorp et al., 2018; Mulholland et al., 2018; Biggio et al., 2019; Hinton et al., 2019; Galaj et al., 2020; Amodeo et al., 2021). During development, pruning and stabilization of dendritic spines in cortical and subcortical regions have been observed in both human (Petanjek et al., 2011) and rodent models of adolescence (Koss et al., 2014; Johnson et al., 2016; Boivin et al., 2018) in a sex and age specific manner (Parker et al., 2020). Both adolescent social isolation and adolescent binge ethanol tend to shift dendritic spine morphology toward more immature spines (thin/filopodia) and fewer mature spines (stubby/mushroom). However, the specific brain regions assessed in most of these studies do not always quantify these dendritic spine alterations in the same brain regions (Tables 1, 2).

Adolescent social isolation alterations to dendritic spine morphology and density vary by brain region (Silva-Gomez et al., 2003). Single housing for 4 months in Thy1-GFP males and females led to fewer stubby spines and more thin spines in pyramidal neurons in mPFC pyramidal layer III, but no changes in mushroom or filopodia-type spines, or total spine number (Medendorp et al., 2018). The changes in spine morphology, however, were accompanied by impaired long-term potentiation (LTP) in single housed mice (Medendorp et al., 2018). Eight weeks after the onset of adolescent social isolation in rats, proximal and distal spine densities were decreased in the mPFC layer III and CA1 of the hippocampus (Silva-Gomez et al., 2003). However, dendritic length was only altered in the CA1 of single housed rats (Silva-Gomez et al., 2003). In the ventrolateral OFC, adult mice with a history of social isolation from PND 31–60 had elevated spine densities and increased PSD-95 at the synaptic puncta, suggestive of failures in age-appropriate spine pruning (Hinton et al., 2019). Notably, re-socialization for 4 weeks was not sufficient to restore the dendritic spine changes (i.e., decreased mushroom spine density) in dentate gyrus granule cells observed following adolescent social isolation in rats (Biggio et al., 2019). Thin or stubby spines were not altered by housing condition (Biggio et al., 2019).

Human postmortem studies and rodent models consistently demonstrate that binge ethanol exposure produces neuroadaptive changes in dendritic spine morphology and density (Ferrer et al., 1986; Harper and Corbett, 1990). However, anatomical, quantification, and dosage differences make comparisons among dendritic spine analyses difficult in adolescent binge ethanol exposure studies. For example, CIE exposure in adolescent rats from PND 28–42 increased density of long thin dendritic spines in layer V pyramidal neurons in the prelimbic layer of the mPFC, suggesting increased immature spines following CIE (Trantham-Davidson et al., 2017). Four cycles of CIE exposure in Thy1-GFP adolescent males (weeks 4–6) led to brain regional alterations in dendritic spine density and morphology (Jury et al., 2017). Spine density was decreased in the infralimbic layer of the mPFC, but was unchanged in the prelimbic layer and the basolateral nucleus of the amygdala (BLA), while adolescent CIE altered morphology such that adult mice had thinner thin spines and wider wide spines, indicating ethanol disruption of the pruning in adolescence (Jury et al., 2017). In the motor and visual cortex, five weeks of CIE in adolescents (PND 22–57) or in adults (PND 91–126) decreased the density of immature dendritic spines in the V1/V2 cortex irrespective of age (Amodeo et al., 2021). However, age-dependent effects were found in the motor cortex where adolescent CIE decreased spine density while adult CIE did not (Amodeo et al., 2021).

A few studies have used less stressful and intense ethanol administration paradigms and exposed animals to ethanol using intraperitoneal injections or gavage (Risher et al., 2015; Mulholland et al., 2018; Galaj et al., 2020; Kipp et al., 2021; Pascual et al., 2021). In general, oral dosing of ethanol in adolescents decreased spine density. Differential effects in age of exposure on dendritic spine density, morphology and excitability in the prelimbic area of the mPFC was found in male rats exposed to intermittent ethanol (4 g/kg i.g.) as adolescents (PND 28–45) or as adults (PND 70–88), 21 days after cessation of ethanol (Galaj et al., 2020). In binge ethanol-exposed adolescent rats, neuronal excitability in prelimbic layer V was increased, and pyramidal neuron spine density was decreased. Contrarily, adult exposed animals showed decreased excitability and increased pyramidal neuron spine density in layer V. There were also age-dependent effects when subclassifying spines into thin (long/thin and filopodial) and non-thin (stubby/mushroom); adolescents had decreased non-thin and increased thin spines, while adults had increased non-thin and decreased thin spines 21 days after ethanol gavage ended (Galaj et al., 2020). These changes were not apparent in layer 2 pyramidal neurons. These results also opposed previous reports of changes in prelimbic layer V (Jury et al., 2017; Trantham-Davidson et al., 2017). Intermittent binge ethanol in male and female adolescent rats (PND 22–55, 5 g/kg, i.g.) did not alter total dendritic spine density or proportion of mature or immature spines in the OFC (Kipp et al., 2021). However, the amount of apical dendrite branching was increased by binge ethanol exposure with sex-dependent differences in where along the dendrite the increased branching was found (Kipp et al., 2021). More consistent findings occur within the hippocampus. Adult rats with a history of adolescent binge ethanol (PND 30–45, 5 g/kg i.g.) had fewer distal dendritic spines in granule cells accompanied by fewer immature/thin spines and stubby spines in the dentate gyrus of the hippocampus (Mulholland et al., 2018). Again, filopodia-type spines were diminished and those remaining were categorized as thin spines (Mulholland et al., 2018). Fragile X mental retardation (*Fmr1*) deletion has been implicated in increased spine density in the hippocampus (Grossman et al., 2010). Its downregulation following ethanol exposure in adolescence may partially explain the alterations to dendritic spines in the hippocampus (Mulholland et al., 2018). In CA1 of adults with a history of adolescent ethanol (PND 30–46, 5 g/kg, i.g.), an increase in thin and filopodia type spines was seen along with a decrease in stubby and mushroom spines, but without a change in total spine density (Risher et al., 2015). This change was accompanied by increased likelihood of LTP at a low stimulus intensity, suggesting that adolescent ethanol increases synaptic plasticity and synaptic immaturity in the hippocampus (Risher et al., 2015). In the granular cells of the dentate gyrus, intermittent binge ethanol in male and female C57BL/6 mice (PND 30–43, 3 g/kg, i.p.) increased the density of thin spines in females and stubby spines in males without a change in the density of mature mushroom-shaped spines, suggestive of dysfunction of synaptic spine pruning and plasticity (Pascual et al., 2021). Together, these data show brain regional and age-dependent effects of binge ethanol or social isolation in adolescence on dendritic spines, suggesting that certain brain regions may be more vulnerable to these effects.

#### Neurogenesis

Though limited, adult neurogenesis occurs in the subventricular zone and subgranular zone of the dentate gyrus of the hippocampus in rodents (Altman, 1962; Alvarez-Buylla and Garcia-Verdugo, 2002). Proliferating cells express transient markers specific to their maturation phases and many of these are used to quantify neurogenesis. The most common markers for quantifying neurogenesis are Ki67 and BrdU, markers of cells undergoing mitosis, and doublecortin (DCX), a marker of immature neurons (Gage and Temple, 2013). The exact mechanisms underlying neurogenesis are currently unknown, but some changes in neurogenesis have been observed after isolation stress and ethanol exposure.

There is limited research into the effects of adolescent social isolation on neurogenic markers, but studies show decreased neurogenesis in the dentate gyrus of the hippocampus in rats and non-human primates. Eight weeks of social isolation (beginning at PND 21) decreased BrdU+, DCX+, and Ki67+ cells in the dentate gyrus of isolated male rats (Biggio et al., 2019), suggesting reduced hippocampal neurogenesis. As discussed above, this result was accompanied by decreased density of mature, mushroom-shaped spines (Biggio et al., 2019). Rats that were socially isolated for 4 weeks in adolescence, then rehoused into groups of 5 did not have these decreases in BrdU+, DCX+, and Ki67+ cells (Biggio et al., 2019). Social isolation for one or three weeks during the transition from adolescence into adulthood in non-human primates decreased the number of BrdU+ cells and a smaller percentage of these cells were co-labeled with DCX suggesting fewer cells committing to neuronal fate (Cinini et al., 2014).

The adolescent brain seems to be more susceptible to alcohol-related decreases in proliferation. Adolescent (PND 28–48, 4 g/kg, i.g.), but not adult ethanol exposed (PND 70–90, 4 g/kg, i.g.) male rats had decreased DCX+ cells in the dentate gyrus, but not the subventricular zone, of the hippocampus (Broadwater et al., 2014). Intermittent ethanol in early to mid-adolescence (PND 28–41, 2 g/kg, i.p.) reduced the number of DCX+ and Ki67+ cells in the dentate gyrus and subgranular zone of adult rats (Sakharkar et al., 2016). Treatment with trichostatin A, an HDAC inhibitor, restored number of DCX+ and Ki67+ cells to that of water controls (Sakharkar et al., 2016). Intermittent binge ethanol (PND 25–54, 5 g/kg, i.g.) in male Wistar rats decreased BrdU and Ki67, markers of cell proliferation, and DCX, Sox2, and Tbr2, neuronal progenitor markers in the dentate gyrus at PND 95, but not at PND 57 (Liu and Crews, 2017). In the dorsal and ventral hippocampus, adolescent binge ethanol (PND 25–55, 5g/kg, i.g.) persistently decreased DCX+ and Ki67+ cells and the number of DCX+ cells was positively correlated with novel object recognition memory (Vetreno and Crews, 2015). Adult rats without a history of binge ethanol were injected with LPS at PND 70, recapitulating the decrease of DCX+ cells in the dentate gyrus, suggesting that neuroinflammation after ethanol administration may underlie the decrease in immature neurons (Vetreno and Crews, 2015). Following traumatic brain injury or stroke, there is a transient increase in neurogenesis, likely an endogenous response to acute insult and inflammation (Arvidsson et al., 2002; Wang et al., 2016). There is reported inflammation after higher doses of ethanol, which could contribute to the decrease in cell proliferation and maturation (Vetreno and Crews, 2015; Liu and Crews, 2017). Because of these confounds, it is difficult to establish a causal relationship between decreased proliferation/immature neurons and cognitive deficits observed in these studies. The benefits of neurogenesis in memory have also been argued, further complicating these findings (Shors et al., 2002).

## Conclusion and Future Directions

Adolescence is a critical developmental period where a number of physiological, cognitive, behavioral, and structural changes in the brain are occurring making this age particularly susceptible to long-term changes in behavior that last into adulthood and increase risk for neurological disease (Figure 1). Social experience during this time is crucial for proper neurodevelopment. Postweaning single housing in rodents is frequently used to model social isolation stress and increases social behaviors, anxiety-like phenotypes and locomotor activity, impairs social and working memory, impairs cognitive flexibility, and increases ethanol consumption in adulthood. Ethanol exposure in adolescence is often modeled during mid- to late- adolescence (∼PND 28–45) and leads to increased social facilitation, impaired working memory, cognitive inflexibility, and also tends to increase drinking in adulthood.

The physiological development of the HPA axis also plays a role to influence brain development and behavioral response to stress. Social isolation and binge ethanol in adolescence leads to glucocorticoid insufficiency and dysregulation of the HPA axis (Montesinos et al., 2016) (Varlinskaya et al., 2016; Hinton et al., 2019), and these experiences may delay or prevent the age-appropriate maturation of the HPA axis. Rodents that experience social isolation or binge ethanol exposure in adolescence also tend to display heightened locomotor activity in some tasks (Bibancos et al., 2007; Varlinskaya and Spear, 2008; McLean et al., 2010; Skelly et al., 2015; Wolstenholme et al., 2017; Medendorp et al., 2018; Wang et al., 2019), including some reports of increased swim speed in the Morris water maze (Pisu et al., 2011a; Wang et al., 2019). Indeed, prior ethanol exposure enhances the locomotor stimulating effects of ethanol (Lopez et al., 2003; Hefner and Holmes, 2007; Stevenson et al., 2008; Melon and Boehm, 2011), which persist for at least 3 weeks after the last ethanol administration (Quoilin et al., 2012, 2014), reminiscent of the heightened locomotor sensitization in adults with history of binge ethanol in adolescence (Quoilin et al., 2012). As tasks to measure anxiety phenotypes are typically performed in a novel area, these increases in locomotor activity may be reflective of the generalized increase in stress reactivity resulting from a lack of habituation to a stressor (Vazquez, 1998; Romeo et al., 2006; Varlinskaya et al., 2016) and dysregulation of the HPA axis.

Synaptic pruning is an active mechanism during adolescent brain development and is critical for encoding experience-dependent plasticity in the brain and may be driving changes in local synaptic plasticity observed after use of addictive substances and stress (Robinson and Kolb, 2004; Runge et al., 2020). Adolescent social isolation or binge ethanol exposure shifts dendritic spine morphology toward more immature (thin/filopodia) and fewer mature spines (stubby/mushroom) (Risher et al., 2015; Jury et al., 2017; Trantham-Davidson et al., 2017; Medendorp et al., 2018; Mulholland et al., 2018; Biggio et al., 2019; Galaj et al., 2020; Amodeo et al., 2021; Pascual et al., 2021), suggesting improper pruning and retainment of more immature dendritic spines. It is possible that certain circuits/brain regions are more susceptible to adaptation, causing morphological changes in response to the rewarding drug-seeking behavior and social stress. Changes to the excitatory/inhibitory balance may also underlie the changes to spines and underlie deficits in memory processes. Social isolation decreases glutamatergic activity and increases GABA_*A*_R in the dentate gyrus (Sanna et al., 2011; Talani et al., 2016) and hippocampus (Pisu et al., 2011a) compared to group housed rodents. Contrarily, social isolation in mid-adolescence increases expression of glutamatergic markers in the mPFC later in adulthood (Lander et al., 2017). Changes in NDMA and glutamate receptors are also a proposed mechanism for alterations in spine density and morphology due to ethanol (Carpenter-Hyland and Chandler, 2007). Structural changes in the prefrontal cortex are somewhat similar following social isolation or ethanol exposure, as both lead to deficits in white matter and markers of reduced myelin (De Bellis et al., 2005; Medina et al., 2008; Makinodan et al., 2012; Bava et al., 2013; Montesinos et al., 2014; Vargas et al., 2014; Vetreno et al., 2016; Wolstenholme et al., 2017; Pfefferbaum et al., 2018; Hinton et al., 2019; Tavares et al., 2019). Together the studies discussed above show that social isolation or binge ethanol in adolescence stunts cortical development, maintaining use of the limbic system and the retention of an adolescent phenotype (Spear and Swartzwelder, 2014; Crews et al., 2016).

Adult neurogenesis is also impaired by adolescent social isolation and binge ethanol. As there is no clear mechanism underlying neurogenesis, nor is there a consensus of it occurring in adult humans (Sorrells et al., 2018), there are questions to both the translatability of rodent neurogenesis research and the consequences of increasing neurogenesis in a developing brain. Improper pruning is already observed in adolescents – both human and rodent – following alcohol exposure (Jury et al., 2017; Hinton et al., 2019; Pascual et al., 2021). However, it is unclear if an increase in neurogenesis would be functionally beneficial or detrimental to an adolescent brain (Scharfman and Hen, 2007). Furthermore, increasing neurogenesis could have off-target effects such as improper integration into local hippocampal circuitry, causing seizure activity (Parent et al., 2006).

It is currently unclear if the mechanisms underlying the ethanol and social stress behavioral effects could be shared, or if they are impacting the developing brain through different pathways or at different points in development to elicit the same behavioral outcome. One critical point is that the differing behavioral effects of adolescent social isolation or binge drinking could be due to the timing of the experience – paradigms often start at varying timepoints. Social isolation frequently begins postweaning at PND 21, while binge ethanol models are typically started on PND 28–30, a week later. In rodent models, this one-week difference could account for some of the differing behavioral effects (i.e., locomotor and novelty-induced hyperactivity and stronger hippocampal-mediated spatial memory deficits in socially isolated mice) and may be due to regional differences in brain maturation. Hippocampal-mediated tasks are typically unaltered by binge ethanol (Seemiller and Gould, 2020) and are more consistently found in socially isolated mice, suggesting that because the hippocampus is further along in development at the beginning of a binge ethanol paradigm, it may be more protected from ethanol’s assault. However, in a detailed study investigating the effects of age of binge drinking onset, Metten et al. (2011) have shown that ethanol exposure on weeks 4–5 show the most dramatic effect of increasing ethanol drinking in adulthood. This would suggest that mid-to late adolescence is more vulnerable to ethanol-inducing behavioral effects than early (PND 21–27) adolescence, while the opposite is true for social isolation. This highlights the importance of studying these two adolescent experiences concurrently.

Sex differences are only beginning to be studied in the behavioral and structural changes induced by social isolation or binge drinking. In the cases where both sexes are included, females do not always respond in the same manner or even the same direction as males. Sex differences in alcohol use begin to develop in adolescence, and continue to impact adult physiology (Peltier et al., 2019). Clinically, women present with symptoms of alcohol-related problems sooner after their first drinking experience (Bradley et al., 1998) and progress through the development of AUDs faster than men (Randall et al., 1999). Female rodents, in general, consume greater amounts of ethanol than their male counterparts (Vetter-O’Hagen et al., 2009; Alfonso-Loeches et al., 2013) and are more sensitive to ethanol-induced neuroinflammation (Alfonso-Loeches et al., 2013; Montesinos et al., 2014). In female rats, social isolation does not consistently lead to increased ethanol intake and preference (Chappell et al., 2013; Skelly et al., 2015; Scott et al., 2020), whereas single housed male rats and (Schenk et al., 1990; Wolffgramm, 1990; Chappell et al., 2013; Skelly et al., 2015) and mice (Advani et al., 2007; Lopez et al., 2011; Sanna et al., 2011) typically increase their intake. In clinical studies, adolescent females (aged 15–18) showed a stronger association of anxiety and depressive symptoms with an earlier drinking onset (Johannessen et al., 2017). Depression symptoms in females during early adolescence was also associated with increased prevalence of problematic drinking in later adolescence (Edwards et al., 2014). These differences may be related to underlying sex differences in cortico-limbic brain development and HPA axis reactivity (Panagiotakopoulos and Neigh, 2014; Roberts and Lopez-Duran, 2019; Premachandran et al., 2020). Similar to other stressors, alcohol leads to an increased stress response and greater levels of ACTH and CORT in adult females compared to males (Rivier, 1993). In adolescence, females may have a higher HPA axis reactivity (Gunnar et al., 2009b; Stroud et al., 2011; Ji et al., 2016) whereas post-pubescent males show greater HPA axis reactivity (Panagiotakopoulos and Neigh, 2014; Liu et al., 2017). Conversely, alcohol misuse may promote the expression of anxiety disorders more frequently in males than females (Falk et al., 2008). These sex-related differences in the cortical signaling in the brain produced by the stress of social isolation and/or repeated ethanol exposures also vary as a function of age, which may have implications for understanding sex differences in the etiology of affective disorders and alcoholism co-morbidity.

In many studies, the search for a causal mechanism of adolescent isolation or binge drinking on structural, molecular, or behavioral effects is focused on only one gene or signaling system. While this information is foundational for guiding future studies, one gene is rarely a causative factor in changing a behavior, and identifying the circuitry involved in these effects may be a more fruitful undertaking. Retrograde and anterograde tracers have been used to identify functional connectivity between brain regions for almost 100 years. This technology has come a long way in the last few decades with the use of viral vectors, DREADDs, optogenetics, and transgenic mice to manipulate specific connections between two brain regions in an anatomically and temporally specific manner. There are some complex and thoroughly designed studies making use of these modern manipulative techniques (Abbas et al., 2018; Hinton et al., 2019; Phillips et al., 2019; Sun et al., 2020). Until 10 years ago, neither the alcohol or the social isolation fields have used circuit manipulation to show necessity of a circuit in a particular social, cognitive, or self-administering behavior (Zhu et al., 2014). Further exploration of the interconnectivity of regions implicated in both paradigms, such as the amygdala, prefrontal cortex, and hippocampus, will contribute to the underlying mechanisms resulting in phenotypic changes.

The studies described above are beginning to elucidate the roles of adolescent binge drinking and social isolation stress in brain connectivity and behavior. However, most of these studies have looked at binge drinking and social isolation stress separately. Moving forward, it is important to study both adolescent isolation and binge drinking in conjunction, as these two behaviors often occur together – human studies have shown that stressful early life events are associated with problematic underage drinking and high alcohol use later in life (Dube et al., 2002; Zaso et al., 2021). Studying the long-term impacts of social isolation and the increased risk for developing an AUD later on in life is especially important in light of the COVID-19 global pandemic. Many United States students during this time participated in distance learning and thus, lost the opportunity for in-person social interaction with their peers during a critical point in adolescent brain development (United Nations Educational Scientific and Cultural Organization [UNESCO], 2021). In Canadian teens that were substance users prior to the COVID-19 pandemic, the number of days they used alcohol or cannabis increased in the first 3 weeks of the pandemic (Dumas et al., 2020). Use patterns shifted to more substances being used in isolation (Dumas et al., 2020), likely due to the constraints of social distancing protocols. Although increased drinking in teens during the pandemic has not always been found (White et al., 2020). As teenagers are now increasingly affected by pandemic-related life changes, such as social isolation and boredom, which are increased risk factors for substance use, animal models of social isolation stress combined with substance use paradigms are even more important to understand the mechanisms underlying these behavioral risks and develop therapeutic treatments.

## Author Contributions

JL: conceptualization, formal analysis, writing – original draft, and writing – review and editing. EB: formal analysis and writing – review and editing. JW: conceptualization, methodology, validation, formal analysis, resources, funding acquisition, writing – original draft, writing – review and editing, supervision, and project administration. All authors: contributed to the article and approved the submitted version.

## Conflict of Interest

The authors declare that the research was conducted in the absence of any commercial or financial relationships that could be construed as a potential conflict of interest.

## Publisher’s Note

All claims expressed in this article are solely those of the authors and do not necessarily represent those of their affiliated organizations, or those of the publisher, the editors and the reviewers. Any product that may be evaluated in this article, or claim that may be made by its manufacturer, is not guaranteed or endorsed by the publisher.
